# Modification of a Two-Part Cancellous Locking Screw: A Pilot Study on Increasing Resistance to Axial Pullout Strength

**DOI:** 10.3390/bioengineering12050444

**Published:** 2025-04-23

**Authors:** Chia-Hao Hsu, Nin-Chieh Hsu, Sung-Yen Lin, Cheng-Chang Lu, Yin-Chih Fu, Hsuan-Ti Huang, Chung-Hwan Chen, Pei-Hsi Chou

**Affiliations:** 1 Graduate Institute of Clinical Medicine, College of Medicine, Kaohsiung Medical University, No. 100, Shih-Chuan 1st Road, Sanmin District, Kaohsiung 80708, Taiwan; ecowarrior.tw@yahoo.com.tw (C.-H.H.); hwan@kmu.edu.tw (C.-H.C.); 2 Department of Orthopedics, Kaohsiung Medical University Hospital, No. 100, Tzyou 1st Road, Sanmin District, Kaohsiung 80756, Taiwan; tony8501031@gmail.com (S.-Y.L.); cclu0880330@gmail.com (C.-C.L.); microfu@ms.kmuh.org.tw (Y.-C.F.); hthuang@kmu.edu.tw (H.-T.H.); 3 Department of Orthopedics, College of Medicine, Kaohsiung Medical University, No. 100, Shiquan 1st Road, Sanmin District, Kaohsiung 80708, Taiwan; 4 Orthopaedic Research Center, Kaohsiung Medical University, Kaohsiung 80708, Taiwan; 5 Regenerative Medicine and Cell Therapy Research Center, Kaohsiung Medical University, Kaohsiung 80708, Taiwan; 6 Department of Internal Medicine, College of Medicine, National Taiwan University, Taipei 100225, Taiwan; chesthsu@gmail.com; 7 Division of Hospital Medicine, Department of Internal Medicine, Taipei City Hospital Zhongxing Branch, Taipei 103212, Taiwan; 8 School of Post-Baccalaureate Medicine, College of Medicine, Kaohsiung Medical University, Kaohsiung 80708, Taiwan; 9 Department of Orthopedics, Kaohsiung Medical University Gangshan Hospital, Kaohsiung 820, Taiwan; 10 Department of Orthopedics, Kaohsiung Municipal Hsiao-Kang Hospital, Kaohsiung Medical University, Kaohsiung 812, Taiwan; 11 Department of Sports Medicine, College of Medicine, Kaohsiung Medical University, Kaohsiung 80708, Taiwan

**Keywords:** screw failure, pull-out failure, two-part locking cancellous screw modification, locking screw, two-part screw, axial pullout resistance, osteoporotic metaphyseal region, biomechanical study

## Abstract

**Background/Objectives:** The pullout failure of conventional locking screws (LSs, screws with a locking mechanism) may occur in patients with osteoporosis, particularly when inserted near joints or across periarticular fractures (e.g., proximal humerus). The two-part locking cancellous screw modification (TP-LCS, screws composed of two parts) in metaphyseal cancellous bone is hypothesized to increase bone purchase and holding power. This study aimed to test the hypothesized advantages of TP-LCS over LSs. **Methods:** An MTS 370 series frame with an axial/torsional load cell was used to test driving torque and axial pullout strength, following ASTM F543-07 standards. The TP-LCS group featured a newly modified screw design made from titanium alloy (Ti6Al4V), while conventional LSs (Synthes) were used for the control group. Statistical significance was assessed for selected comparisons relevant to the research objectives, including driving torque and axial pullout strength. **Results:** The driving torque test showed that TP-LCS had a significantly higher maximum insertion torque (4.9 ± 0.4 N·cm) compared to LSs (4.2 ± 0.4 N·cm) (*p* = 0.0269), although no significant difference was found in maximum removal torque (*p* = 0.1046). The axial pullout test revealed that TP-LCS had significantly higher pullout strength (223.5 ± 12.2 N) compared to LSs (203.5 ± 11.5 N) (*p* = 0.0284). Failure during the axial pullout test often involved cracking of the test block material around the screw threads, causing the screw to pull out. **Conclusions:** These results support the hypothesis that TP-LCS may offer improved axial pullout resistance compared to LSs, making it a potentially beneficial modification for LSs in osteoporotic metaphyseal regions or near joints. This study provides biomechanical insights into the advantages of the modified screw design over conventional LSs.

## 1. Introduction

Non-locking plates rely on the frictional forces between the bone and the plate to resist shear forces. On the other hand, locking plates convert shear stress into compressive stress at the screw–bone interface, functioning as a unified beam structure. Furthermore, in a locking plate system, the total fixation strength is the cumulative holding power at all screw–bone interfaces, unlike non-locking plates, which depend on the friction generated by screw compression for stability [1,2]. Particularly over the past twenty years, locking plate–screw systems have become progressively more popular. However, they come with many pitfalls and limitations, and have experienced a significant number of failures [3,4]. The fixation of the plate to the bone depends on screws, and several designs have been created to ensure a stable and rigid angle between the plate and the screws [5]. Locking plates function as internal fixators on the cortical surface, evenly distributing the loading forces along the fracture line to all screws simultaneously. This mechanism is similar to the way external fixators stabilize fractures; however, locking plates provide the advantage of internal fixation, offering improved stability and a reduced risk of displacement.

Locking plate failures can stem from either bone-related or implant-related factors [4], with screw pull-out being a primary cause of failure in such procedures [3,6]. Notably, screw anchorage may be further compromised by thermal and mechanical damage to the bone incurred during drilling, which negatively affects the integrity of the bone–screw interface [7,8]. In traditional locking plates, poor attachment to osteoporotic cancellous bone is a common problem due to the reduced ability of the screws to anchor securely in the softer, more porous bone structure, compounded by the small thread pitch and shallow thread depth. Moreover, studies have demonstrated that inappropriate drilling parameters can exacerbate this issue by increasing local bone temperature and inducing additional mechanical damage, thereby elevating the risk of screw loosening or pull-out [7,8]. Internal fixation with a locking plate has become a common approach for stabilizing unstable proximal humeral fractures. However, despite its success, this treatment is associated with a complication rate as high as 49% [9,10], most of which is related to pullout failure, with revision surgeries being necessary in as many as 25% of cases [11,12,13].

In the treatment of osteoporotic metaphyseal areas, employing cancellous screws may be more advantageous. Due to their larger thread pitch and greater thread depth, cancellous screws may provide better attachment to osteoporotic bone. However, while the pull-out issue is often cited as a potential complication for locking screws (LSs) in osteoporotic bones [3,6], cancellous screw-type LSs are not included in many FDA-approved locking plate systems.

The hypothesis of our study is that the usage of a cancellous screw modification of conventional LSs provides better bone purchase and bone holding power and improves the pull-out issue. Although some previous studies have found that the pull-out strength of traditional non-locking cancellous screws is greater compared to cortical and other types of non-locking screws in the cancellous bone region [14,15], modifications in locking systems, particularly in newer designs, have not been well studied to date. As a result, we developed a prototype of a novel cancellous screw modification of conventional LSs with an additional locking nut (two-part locking cancellous screw, TP-LCS). The aim of this study is to evaluate and compare the screw driving torque and pull-out strength of our novel TP-LCS model with those of an FDA-approved classical LS in a simulated synthetic bone model.

## 2. Materials and Methods

### 2.1. Screw Design and Rationale

**Figure 1:**
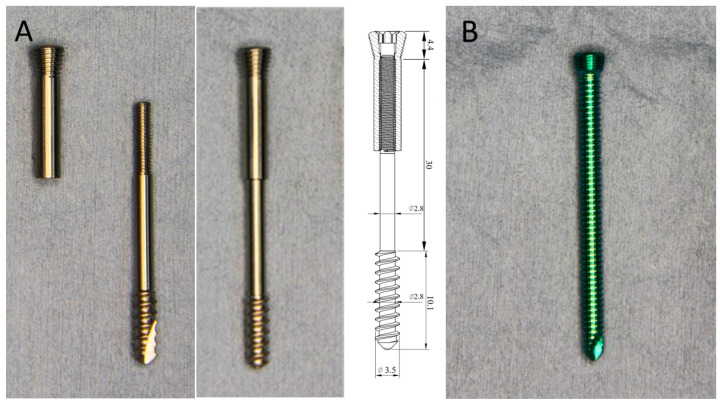
(**A**) Left: the two-part screw consists of two components: a main screw (cancellous screw modification) and a locking nut. The schematic diagram illustrates the internal structure of the combination, with proportions not to scale. It shows that the two parts are connected by threads. (**B**) Right: conventional locking screw (Synthes, Paoli, PA, USA). For detailed specifications of both screws, please refer to Section 2.2.

The concept of the novel TP-LCS was developed by the authors. It has two components: a main screw (cancellous screw modification) and a locking nut. The TP-LCS is a typical partially threaded cancellous screw that can potentially serve as a lag screw (lag screw by design). It functions essentially as a lag screw with a locking nut (Figure 1A), which has the same locking threads on the locking nut as conventional LSs (Figure 1B). The locking nut serves to provide the cancellous screw-type main screw with additional locking ability via a locking head. This modification may ensure that the cancellous screw-type main screw is locked onto the plate in the same way as a conventional LS.

This modified screw design consists of two parts connected by threads to achieve full combination. The distal part has typical cancellous screw-type threads, which are partially threaded, while the conventional LS is fully threaded. Each part has a driver recess at the screw head for independent rotational control by a screwdriver. The main screw part can be inserted and removed using a smaller screwdriver. The locking nut can be inserted and removed using a larger standard Stardrive^®^ screwdriver (Synthes, Paoli, PA, USA), which is intentionally designed to match the conventional LS in this study and is compatible with a standard Stardrive^®^ recess. The locking nut of the TP-LCS can be fully tightened to secure both parts together as a single unit. When removed, both components can be pulled out together as one integrated piece.

Once the TP-LCS is fully integrated and tightened into a single unit, its function becomes equivalent to that of a standard LS. It can then be inserted into the locking hole on the locking plate using the same Stardrive^®^ screwdriver typically used for conventional LSs. The only difference is that the distal end of the screw features a cancellous screw thread.

### 2.2. Biomechanical Analysis

The experimental group consists of TP-LCS samples, which were designed to have a locking head compatible with Locking Compression Plates (Synthes, Paoli, PA, USA) and were manufactured by an orthopedic implant manufacturer in Taiwan (BAUI Biotech Co., Ltd., Taipei, Taiwan). The screw head of the TP-LCS samples was designed to correspond with the standard LS head (Figure 1A). The distal threads are 10.1 mm in length. The core diameter and thread diameter are 2.8 mm and 3.5 mm, respectively. Therefore, the thread depth is 0.7 mm (3.5 mm − 2.8 mm). The thread pitch is 1.6 mm. The overall screw length is 44.5 mm. The head length and shank length are 4.4 mm and 30 mm, respectively. The screw is made from surgical grade titanium alloy (Ti6Al4V) and has no surface treatment.

For comparison, we aim to use an FDA-approved, widely used LS from a global firm as the control. The control group consisted of conventional LSs (Synthes, Paoli, PA, USA). These are one-piece, fully threaded LSs (Figure 1B). The screw head features a standard AO locking screw head, as does the thread. The core diameter and thread diameter are 2.9 mm and 3.5 mm, respectively. Therefore, the thread depth is 0.6 mm (3.5 mm − 2.9 mm). The thread pitch is 0.72 mm. The overall screw length is 45.2 mm. The head length is 3.2 mm. The screw is made from titanium alloy (TiAl6 Nb7).

For the purpose of this study, two types of tests were performed: driving torque and axial pullout strength. The experiments were conducted using an MTS 370 series frame, equipped with an axial/torsional load cell (model 662.20H-05), which provides axial and torsional capacities of 25 kN and 250 N·m, respectively. The loading conditions and test method follow ASTM F543-07 [16].

For the axial pullout tests, the two screw parts were assembled and fully tightened as shown in Figure 1A. During the driving torque tests, only the main screw part was used to measure the insertion and removal torque.

**Figure 2:**
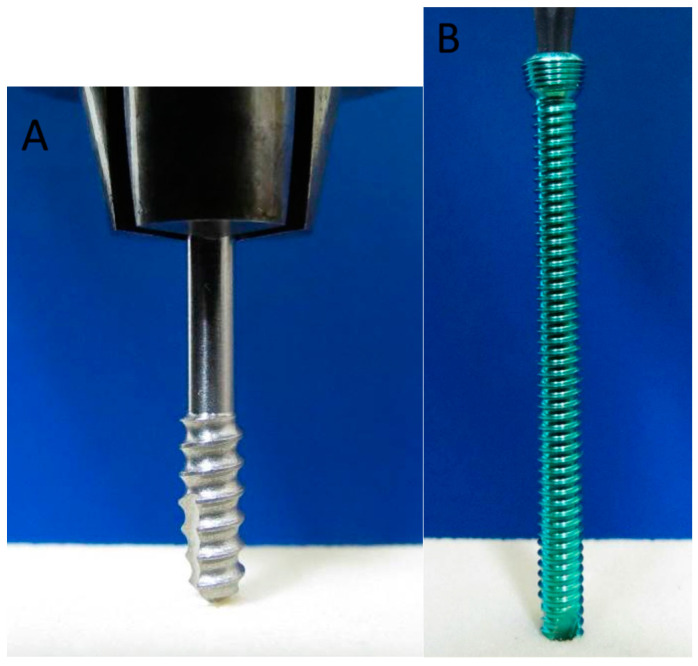
The test setup for the driving torque tests: (**A**) Left: Main screw part (cancellous screw modification) of the two-part screw. (**B**) Right: Conventional locking screw (Synthes, Paoli, PA, USA).

For the driving torque tests, five screws from each group were tested. The test setup is shown in Figure 2, with “Solid Rigid Polyurethane Foam” used as the test block. The test blocks were manufactured using Sawbones^®^ (Pacific Research Laboratories, Inc., Vashon, WA, USA) and identified by item number 1522-03. The material composition consisted of polyurethane foam with a density of 20 pcf (0.32 g/cc), conforming to ASTM F1839-08 [17]. The foam’s tensile strength was 5.6 MPa, compression strength was 8.4 MPa, and shear strength was 4.3 MPa [18]. The polyurethane foam (density 20 pcf) used in our study approximates the mechanical properties of metaphyseal bone, which has a density range of 0.2–0.4 g/cc and compressive strength of 2–10 MPa. The foam’s compressive strength of 8.4 MPa is similar to that of metaphyseal bone, while its tensile and shear strengths are slightly higher than natural bone but still within a reasonable range. Thus, the foam serves as a reasonable approximation for the metaphyseal region in biomechanical studies, with some differences in strength compared to natural bone.

The pilot hole was drilled into the test block (2.8 mm (TP-LCS) and 2.9 mm (LS) in diameter and 20 mm in depth). An axial load of 1.14 kgf was applied throughout the entire test, which was maintained using the MTS 370 series frame equipped with an axial/torsional load cell. The screw was then inserted into the test block for four revolutions (about 8 mm (TP-LCS) and 7 mm (LS) in depth). The maximum torque during insertion and removal was recorded.

**Figure 3:**
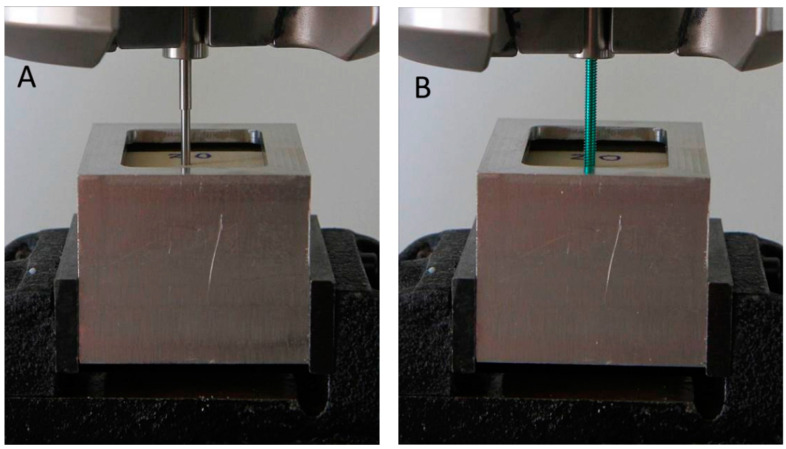
The test setup for the axial pullout tests: (**A**) Left: two-part screw (both parts assembled and fully tightened). (**B**) Right: conventional locking screw (Synthes, Paoli, PA, USA).

For the axial pullout tests, five screws from each group were tested. The test setup is shown in Figure 3. The grip span was set to 40 mm, and the screw was inserted into the test block to a depth of 10 mm, with the total thickness of the test block being 40 mm. The same type of test block was used as in the driving torque tests.

We chose the minimum number (five) of test samples that was just sufficient for statistical analysis. Previous studies have shown that sample sizes estimated using one-dimensional power procedures vary according to the characteristics of the dataset, typically requiring small-to-moderate sample sizes of approximately 5–40 to achieve target powers of 0.8 for reported one-dimensional effects. Datasets with large effect sizes (>1) achieved >0.8 power with small sample sizes (5–10) [19]. To justify our choice of sample size, we refer to the effect sizes calculated from the results for insertion torque, removal torque, and axial pullout strength, which were 1.75, 1.18, and 1.69, respectively, indicating that small sample sizes are adequate for statistical analysis.

The study presented descriptive statistics of the data, including mean and standard deviation. The intention was to use five independent screws for each type, conducting five repeat tests to obtain the most representative average values, with no specific pairing of TP-LCS 1 and LS 1 for comparison. Therefore, an independent-samples *t*-test was applied, with a *p*-value of <0.05 considered statistically significant. The Shapiro–Wilk test, suitable for small sample sizes, showed *p*-values for insertion torque, removal torque, and axial pullout strength of 0.99, 0.31, and 0.92, respectively. As all *p*-values exceed 0.05, there is no evidence of non-normal distribution, suggesting the datasets follow a normal distribution. To provide additional validation, we also performed the Mann–Whitney U test as a non-parametric alternative. The results for insertion torque (*p* = 0.0366), removal torque (*p* = 0.1443), and axial pullout strength (*p* = 0.0366) remained consistent, with significant differences observed for insertion torque and axial pullout strength. These statistical analyses, combined with the observed large effect sizes, support a confidence level of at least 95% for the reported findings, indicating the high reliability and reproducibility of the results.

## 3. Results

### 3.1. Driving Torque

Five screws were tested. The results of insertion and removal torques are shown in Table 1. The negative values represent the counterclockwise torque (removal torque). The mean maximum insertion torque for the TP-LCS and LS was 4.9 ± 0.4 N·cm and 4.2 ± 0.4 N·cm, respectively. TP-LCS had a significantly 16% higher mean maximum insertion torque than LS (*p* = 0.0269). The mean maximum removal torque for the TP-LCS and LS was −3.8 ± 0.2 N·cm and −3.5 ± 0.3 N·cm, respectively. However, there was no significant difference in the maximum removal torque between the TP-LCS and LS. The data were consistent between different screws, with a small standard deviation (0.2–0.4 N·cm). The removal torque was slightly lower than the insertion torque. In the TP-LCS, the mean maximum removal torque decreased by 22% compared to the mean maximum insertion torque (−3.8 vs. 4.9), while in the LS, the mean maximum removal torque decreased by 16.7% compared to the mean maximum insertion torque (−3.5 vs. 4.2). The decrease magnitude was similar between the two groups.

### 3.2. Axial Pullout Test

**Figure 4:**
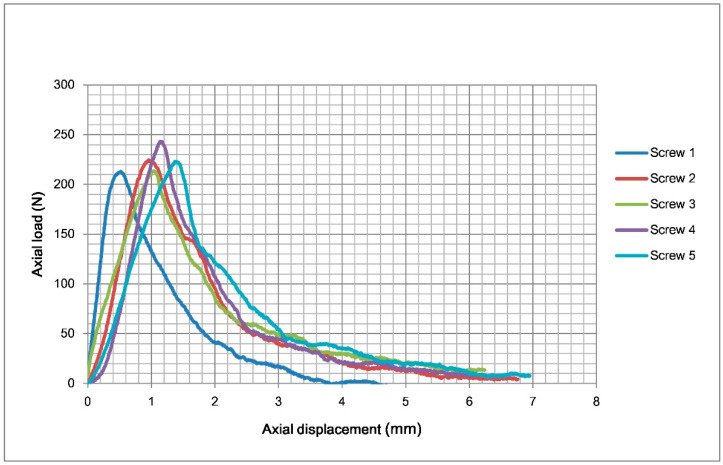
The load–displacement curves of the five tested TP-LCS samples in the axial pullout tests. TP-LCS: two-part locking cancellous screw.

**Figure 5:**
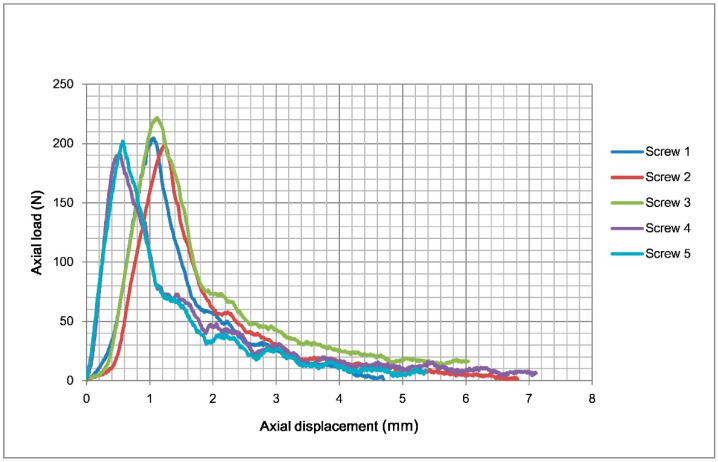
The load–displacement curves of the five tested LS in the axial pullout tests. LS: locking screw (Synthes, Paoli, PA, USA).

Five screws were tested. The results of the axial pullout test are shown in Table 1. The mean axial pullout strength of the TP-LCS and LS was 223.5 ± 12.2 N and 203.5 ± 11.5 N, respectively. The mean axial pullout strength of the TP-LCS is approximately 10% greater than that of the LS. The TP-LCS had a significantly higher axial pullout strength than the LS (*p* = 0.0284), which may also indicate that TP-LCS has significantly greater resistance to pullout from the test block. The diagram of load–displacement curves for the five tested TP-LCS samples is shown in Figure 4, and Figure 5 presents the load–displacement curves of the five tested LSs.

**Figure 6:**
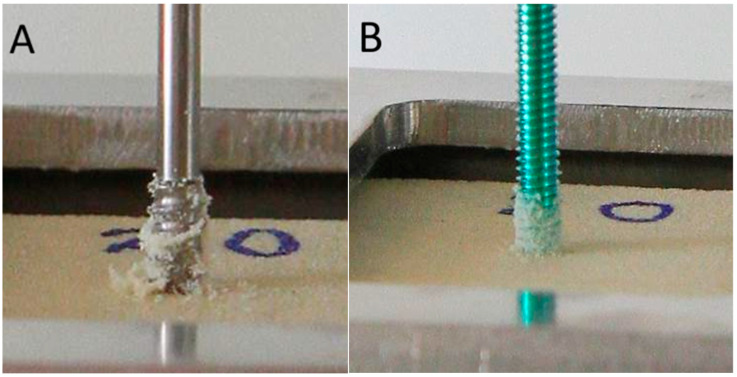
The failure mode of the axial pullout test: (**A**) Left: two-part screw (cancellous screw modification). (**B**) Right: conventional locking screw (Synthes, Paoli, PA, USA). The photographs showed that the cancellous screw modification carried out and retained more material from the test block after being pulled out, implying that it may have greater bone holding power or bone purchase.

The classic failure mode is when the material of the test block around the screw thread cracks, causing the screw to pull out directly from the test block (Figure 6). In this photograph, it is clearly shown that the cancellous screw modification carried out and retained more material from the test block after being pulled out, implying that it may have greater bone holding power or bone purchase.

## 4. Discussion

Screw pull-out is a recognized factor contributing to the failure of locking constructs in osteoporotic bones and metaphyseal fractures [3,6], including proximal humerus fractures [9,10]. This complication may lead to treatment failure and necessitate revision [11,12,13]. However, cancellous screws are not typically used in a locking configuration with locking plates [4]. Hypothetically, incorporating locking cancellous screws in osteoporotic metaphyseal regions could reduce the risk of screw pull-out. To assess the effectiveness of this approach, we aimed to develop a new TP-LCS that modifies the LS with a cancellous screw system and compare it to a conventional FDA-approved LS in a simulated synthetic bone model, focusing on the bone purchase and holding strength of their distal screw threads.

In locking plates, shear forces are transformed into compressive forces at the screw–bone interface, with the screws collectively resisting the deforming forces on the bone, acting as an integrated structure that distributes the forces evenly across all screws [20].

Locking plates exhibit minimal elasticity, which can cause micro-movement at the fracture site, especially in bridge plating. However, this is generally limited to the diaphyseal region. This micro-movement stimulates callus formation, leading to secondary fracture healing (endochondral healing) [21]. In the metaphyseal region, where bridge plating is less applicable, previous studies have indicated that the rigid fixation provided by locking plates may delay secondary fracture healing by hindering callus formation [20,22]. In periarticular fractures, this delay can be as high as 19% [23,24]. As a result, several less rigid locking designs have been developed over the past two decades to reduce the stiffness of the construct and promote secondary healing [25,26], including the use of semi-rigid screws, such as far cortical locking [27,28,29,30,31] and dynamic locking screws [32,33,34,35,36,37].

Nevertheless, micro-movement within locking plates must be carefully controlled to ensure it is restricted to a range at the screw–bone interface and occurs primarily in the axial direction. Excessive micro-movement along the screw’s axis should be avoided to prevent gradual loosening in the direction parallel to the screws, which may, ultimately, lead to pull-out, cut-out, or perforation. Any movement at this interface can exacerbate bone–screw attachment issues, leading to instability. Therefore, a semi-rigid design should offer better screw–bone purchase, enhanced bone holding power, and improved pull-out resistance, counteracting the potential increased risk of pull-out, cut-out, or perforation. The critical factor in the locking plate–screw system is the strength of the attachment between the screws and the bone. If the screws are securely anchored to the bone in both systems, the plate–screw fixation can function as intended [4]. This highlights the importance of increasing the pull-out resistance of the locking screws.

When locking occurs without delay, the screw smoothly progresses into the bone and preserves the integrity of the threads it creates for attachment. On the other hand, delayed or non-smooth locking may cause damage to the existing threads formed by the screw in the bone, potentially weakening the holding strength at the bone–screw interface and resulting in unstable micro-movement. This happens when the screw rotates before it fully engages with the bone or before the screw fully engages with the threads of the locking hole. This phenomenon likely explains the significant variation in the holding power of screws in traditional locking plates. Therefore, the locking nut design in the TP-LCS used in our study may offer the potential benefit of achieving a secure lock without causing additional rotation of the main screw. However, the optimal length of the locking nut or its optimal combination mechanism (such as all-threaded, restrictive sliding, or other secure methods) has yet to be determined in future studies. This pilot study does not include an evaluation of the influence of the locking nut, and we have made every effort to eliminate the interference of this component as a confounding factor in the study.

As a result of the in vitro pull-out test, the TP-LCS prototype we introduced demonstrated significantly better bone purchase and more stable outcomes in a simulated synthetic bone model compared to the conventional LS. The mean axial pull-out strength of the TP-LCS is approximately 10% greater than that of the LS. This improvement can be attributed to the larger thread pitch (1.6 mm vs. 0.72 mm) and deeper thread depth (0.7 mm vs. 0.6 mm) of the cancellous screw modification in the TP-LCS compared to the conventional LS. Our findings support the hypothesis that the TP-LCS may offer enhanced axial pull-out resistance relative to the LS. It is reasonable to speculate that multiple TP-LCSs may have a synergistic or additive effect. Patients with osteoporotic metaphyseal fractures, such as proximal humerus fractures, may hypothetically experience fewer complications or revision surgeries with this novel TP-LCS model, as it could address one of the primary causes of locking plate failure—screw pull-out. However, these potential benefits remain speculative and require further clinical validation before any definitive conclusions can be drawn.

The insertion of the TP-LCS is technique-demanding and requires careful attention. Based on our preliminary experience using the TP-LCS using Sawbones, we acknowledge the learning curve involved. Key factors for successful insertion include ensuring the pre-drilling or reaming of the locking nut area is sufficiently enlarged and accurately estimating the screw length. Fluoroscopic guidance during screw placement can be beneficial, and shortening the locking nut length may improve ease of use. However, the optimal locking nut length and the best method for combining the components still require further investigation. Thus, while the TP-LCS shows potential benefits, further optimization and research are necessary to refine the technique.

Future research should focus on a comprehensive evaluation of the locking nut’s design parameters, including its length, threading mechanism, and ease of insertion under clinical conditions. Comparative studies using different locking mechanisms (e.g., full-threaded, partial-threaded, and sliding designs) should be conducted to determine the optimal configuration. Additionally, finite element analyses and cadaveric studies could provide deeper insights into stress distribution, screw–bone interaction, and failure mechanisms in more realistic anatomical settings. Large-scale mechanical testing with greater sample sizes and more clinically representative test setups, such as multi-screw and plate constructs, is also warranted. Ultimately, clinical trials will be essential to evaluate the efficacy and safety of TP-LCS in vivo and assess its ability to reduce revision rates in patients with osteoporotic metaphyseal fractures. Furthermore, future research should investigate the thermal effects associated with pre-drilling or reaming during TP-LCS insertion, including the potential risk of thermal necrosis, particularly in cadaveric or clinical settings where bone vitality is a concern.

In summary, this study presents a novel concept for improving locking screw bone purchase in the treatment of osteoporotic metaphyseal fractures, such as proximal humeral fractures, along with new insights into the biomechanics of pull-out resistance of such a cancellous screw modification, which can be applied in the development of next-generation implants.

Our study has several limitations. First, this pilot study focused solely on investigating and comparing the biomechanical properties of the most critical screw threads, such as driving torque and axial pull-out strength, between the distal threads of the two screws. It did not evaluate the influence of the locking nut. We made every effort to exclude any confounding factors related to the locking nut to maintain the accuracy and scientific rigor of this study. Second, the study results are limited to the current secure threaded design for the two-part combination of the TP-LCS and cannot be applied to other types of combination designs, such as restrictive sliding. The optimal combination needs to be determined through future studies. Nevertheless, no cracks occurred in the current combination design during the axial pull-out tests. Third, only five samples per group were tested, which may limit the generalizability of the results. Fourth, we tested only the properties of a single screw, which may not be directly applicable to the complex and intricate distribution of screws in plate configurations. However, it can be inferred that multiple TP-LCSs working together would produce even better pull-out resistance, with an expected synergistic effect, since a single TP-LCS has already shown significant superiority over a single LS in terms of pull-out resistance. Nonetheless, this single screw comparison offers a more scientifically fair, unbiased, and credible approach, while minimizing confounding factors related to screw position or trajectory, thereby strengthening the reliability of this study. Finally, the use of polyurethane foam as a synthetic bone model, while reasonable for simulating metaphyseal bone, does not fully replicate the biological and structural complexity of real bone, such as porosity and vascularization.

## 5. Conclusions

In conclusion, the in vitro pull-out tests demonstrated that the novel TP-LCS model exhibited significantly stronger bone attachment compared to conventional LS. The results support the hypothesis that TP-LCS provides superior axial pull-out resistance, making it a promising modification of LS, with potential benefits in osteoporotic metaphyseal regions or near joints. However, it is important to note that these findings are based on in vitro data, and clinical confirmation is required to validate the potential advantages of this modification in real-world settings. Future studies, including cadaveric testing and clinical trials, will be crucial to confirm the in vivo effectiveness of the TP-LCS, evaluate its long-term outcomes, and assess its clinical relevance in improving screw fixation. This study provides valuable biomechanical insights into the benefits of the modified screw design over conventional LS, highlighting its ability to improve pull-out resistance in clinical settings.

## Figures and Tables

**Table 1:** A comparison of TP-LCS and LS results obtained from the driving torque test and axial pullout test.

	TP-LCS		LS		*p*-Value ^#^	TP-LCS/LS	Raw Data of TP-LCS and LS				
Property	Mean	SD	Mean	SD			Screw 1	Screw 2	Screw 3	Screw 4	Screw 5
**Driving torque test**											
Max. insertion torque (N·cm)	4.9	0.4	4.2	0.4	**0.0269**	TP-LCS LS	5.0 4.6	5.0 3.6	5.3 4.1	4.7 4.5	4.3 4.2
Max. removal torque (N·cm)	−3.8	0.2	−3.5	0.3	0.1046	TP-LCS LS	−3.5 −3.5	−4.0 −3.5	−3.8 −3.9	−3.9 −3.1	−3.7 −3.5
**Axial pullout test**											
Axial pullout strength (N)	223.5	12.2	203.5	11.5	**0.0284**	TP-LCS LS	213.1 204.7	224.6 197.8	213.5 221.9	243.2 191.1	223.1 201.8

Abbreviations: SD, standard deviation; TP-LCS, two-part locking cancellous screw; LS, locking screw (Synthes, Paoli, PA, USA). ^#^ Independent samples—*t*-test was used. Bold indicates statistical significance.

## Data Availability

The original contributions presented in the study are included in the article, further inquiries can be directed to the corresponding author.
